# Isolation, Structural Elucidation, Antioxidant and Hypoglycemic Activity of Polysaccharides of *Brassica rapa* L.

**DOI:** 10.3390/molecules27093002

**Published:** 2022-05-07

**Authors:** Wenyang Cao, Chenxi Wang, Xiayidan Mayhesumu, Le Pan, Yan Dang, Abulimiti Yili, Aytursun Abuduwaili, Sanawar Mansur

**Affiliations:** 1 College of Chemistry and Chemical Engineering, Xinjiang Agricultural University, Urumqi 830052, China; caoziye88@163.com (W.C.); w18129196969@163.com (C.W.); xiayidanms@sina.com (X.M.); inmail911@sina.com (L.P.); mg20000330@163.com (Y.D.); 2 Key Laboratory of Plants Resources and Chemistry of Arid Zone, Xinjiang Technical Institute of Physics and Chemistry, Chinese Academy of Sciences, Urumqi 830011, China; abu@ms.xjb.ac.cn

**Keywords:** *Brassica rapa* L., MUAE, polysaccharides, antioxidant and hypoglycemic activities, DPPH, α-glucosidase, α-amylase

## Abstract

The aim of this study was to investigate the effects of microwave ultrasonic-assisted extraction (MUAE) on the content, structure, and biological functions of *Brassica rapa* L. polysaccharide (BRP). Response surface methodology (RSM) was used to optimize the parameters of MUAE, and it obtained a polysaccharide with yield of 21.802%. Then, a neutral polysaccharide named BRP-1-1 with a molecular weight of 31.378 kDa was isolated and purified from BRP using DEAE-650 M and Sephadex G-100. The structures of the BRP-1-1 were elucidated through a combination of FT-IR, GC-MS, NMR, and methylation analysis. The results showed that BRP-1 consisted of mannose (Man) and glucose (Glu) in a molar ratio of 7.62:1. The backbone of BRP-1-1 mainly consisted of →6)-α-D-Glup-(1→4-β-D-Glup-(1→2)-α-D-Manp-(1→2)-α-D-Glup-(1→, the branch was [T-α-D-Manp-(1]_n_→. BRP-1-1 intervention significantly inhibited α-glucosidase activity; an inhibition rate of 44.623% was achieved at a concentration of 0.5 mg/mL. The results of the in vitro biological activity showed that BRP-1-1 has good antioxidant and hypoglycemic activity, suggesting that BRP-1-1 could be developed as a functional medicine.

## 1. Introduction

*Brassica rapa* L. is part of the Cruciferae Brassica family, and is widely distributed in mainland China; local residents call it “Chamaguer” or “Man Jing” in the Xinjiang Uygur Autonomous Region, China. This plant is extensively used in medicine and food because it helps with digestion, diuresis, and cough suppression [1]. The active ingredients in *Brassica rapa* L. have been documented to principally include saponins, flavonoids, polysaccharides, alkaloids, amino acids, and proteins [2]. Polysaccharides act as important bioactive components, and have gradually become a research hotspot in medicine and nutrition in recent years because of their health-promoting effects [3,4]. Studies have shown that BRP has numerous biological functions, including immune regulation, anti-hypoxia, anti-tumor, anti-oxidation, and anti-fatigue activities [5,6,7,8,9].

Extraction methods can result in large differences in polysaccharide contents, structures and biological functions [10,11]. Ultrasound-assisted extraction (UAE) is an extraction technique that disrupts the plant cell wall structure through ultrasonic treatment, resulting in the faster release of polysaccharides; however, it has weak thermal effects and takes a long time to reach the desired temperature levels during the extraction process [12,13]. Microwave ultrasonic-assisted traction (MUAE) is an improvement based on the UAE method, with advantages of a short consumption time and low extraction temperature; it has been demonstrated to be an effective novel technique for polysaccharide extraction [14,15]. However, microwaves may alter the chemical structure and biological activity of polysaccharides [16]. There are currently no relevant studies on the extraction process and product properties of BRP using MUAE.

In this experiment, the response surface methodology (RSM) was used to optimize the extraction parameters (ratio of water to material, microwave-ultrasound time, extraction temperature and ultrasound power) of MUAE. The physicochemical parameters and major chemical structure of BRP-1-1 were determined through NMR and methylation after further separation and purification. Finally, the antioxidant and hypoglycemic activity of BRP-1-1 was tested in vitro. To our knowledge, this is the first time MUAE has been used to extract polysaccharides from *Brassica rapa* L., and the structure and biological applications of BRP fractions have been thoroughly investigated. These results may provide a reference for the integrated use of BRP polysaccharides in health products.

## 2. Results and Discussion

### 2.1. Single-Factor Experiments Analysis

**Figure 1:**
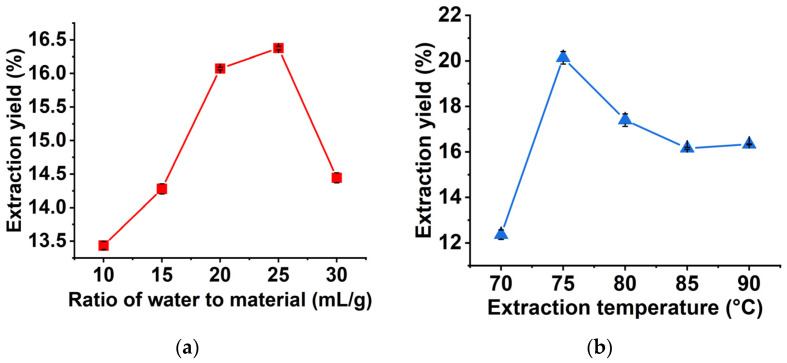

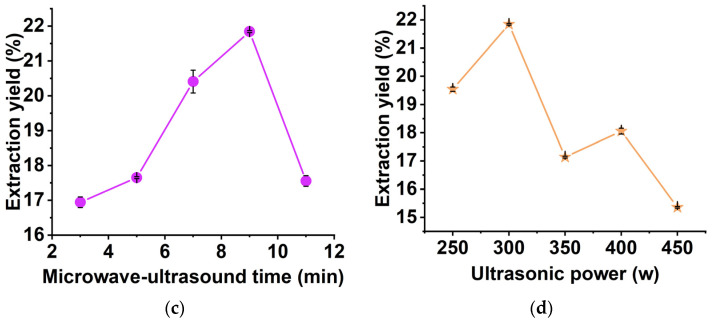
Effect of the single factor on the BRP yield. (**a**) Ratio of water to material (mL/g); (**b**) extraction temperature (°C); (**c**) microwave-ultrasound time (min) and (**d**) ultrasound power (w).

Through experiments with controlled variables, we found that the BRP yield reached a maximum (16.377%) when ratio of water to material was 25:1 mL/g (Figure 1a), the dissolution of BRP rate had reached equilibrium, and the continuous addition of extractant could not produce more polysaccharides, but might lead to the loss of polysaccharides [17]; the BRP yield reached a considerable value (20.130%) at 75 °C, after which the yield gradually decreased (Figure 1b), and the high temperature and the obvious increase in water evaporation may have affected the dissolution of polysaccharides [18]; the yield of BRP reached the maximum (21.843%) at 9 min (Figure 1c), and then began to decline sharply, and the structures and properties of BRP polysaccharides changed (degradation to monosaccharides) under prolonged microwave sonication, causing this result [19]. We also found that the highest BRP yield (21.843%) was obtained at 300 W of ultrasound power (Figure 1d), after which it started to decrease. Higher ultrasound power reduced the cavitation phenomenon, which caused insufficient bubble collapse [20], resulting in a decrease in polysaccharide yield.

Therefore, we set the ratio of water to material of 25 mL/g, extraction temperature of 75 °C, microwave-ultrasound time of 9 min and ultrasound power of 300 W as the center point of RSM experiments.

### 2.2. Response Surface Analysis

#### 2.2.1. Optimization of Extraction Parameters by RSM

As shown in Table S1 (See Supplementary Materials), 29 runs were performed by combining four single-factor parameters (A, B, C and D); the BRP content ranged from 14.750% to 21.873%. The model data were analyzed by regression fitting, and the quadratic multiple regression equations of A, B, C and D with the BRP content (Y) were obtained as follows:

Y = 21.87 + 0.13A + 0.89B + 0.55C + 0.26D + 0.18AB − 0.45AC + 1.24AD + 1.47BC + 0.23BD − 0.75CD − 1.09A^2^ − 3.57B^2^ − 1.39C^2^ − 2.09D^2^ (1)

The analysis of variance (ANOVA) results of the secondary regression model are shown in Table S1 (See Supplementary Materials). The model F = 103.48, *p* < 0.0001 indicated that the equation obtained from this model reached a highly significant level. The correlation coefficient R^2^ = 0.9405 and the adjusted correlation coefficient R^2^_Adj_ = 0.8811 indicated that the experimental and predicted values of the equation had a high correlation [17,18,19,20]. Meanwhile, the F = 8.25 (*p* = 0.82 > 0.05) was a misfitting term, which indicated non-significance and that the experimental error caused by it was small. In addition, the coefficient of variation (C.V) was an important indicator to evaluate the repeatability of the model. C.V % = 4.150% (C.V < 5%), indicating good model repeatability. Finally, the factor significance analysis of the model coefficients showed that *p* < 0.05 for B, C, AD, BC, A^2^, B^2^, C^2^, and D^2^, showing that these factors had a significant effect on the yield of BRP. In addition, because the magnitude of F value reflects the strength of the influence of each factor on the BRP yield, the order of the influence of each factor on the yield is: B > C > D > A. In summary, the proposed model has high accuracy and credibility and can be used for BRP extraction, analysis and prediction.

#### 2.2.2. Analysis of Response Surface

**Figure 2:**
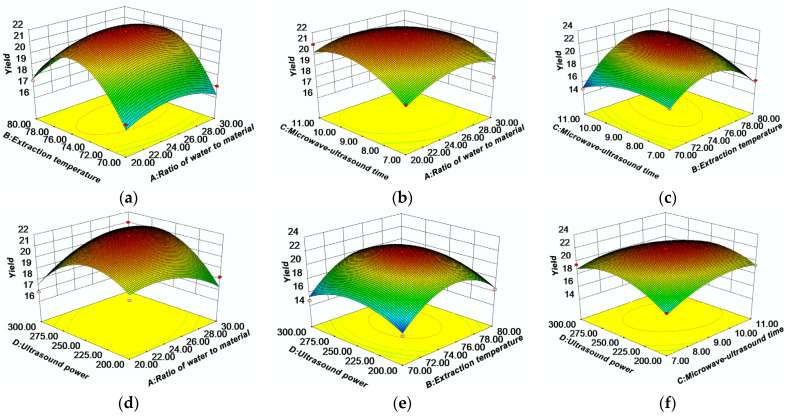
The response surface (3D) shows the effect of each variable (**a**–**f**) on the BRP content.

The interactions between the factors and their effects on the BRP yields are shown in Figure 2. It is known that the interactions between the factors and their influence on the BRP yield can be determined from the shapes of the three-dimensional (3D) response surface plots and contour plots. The steeper the 3D response surface plots, the higher the influence of the factors on the BRP yield. In addition, if the contour plot is elliptical, it indicates a significant interaction between the factors. As shown in Figure 2c,d, the 3D plots are quite steep and the contour plots are elliptical. This indicates that the interaction between AD and BC is significant and affects the polysaccharide yield to a higher extent. This is consistent with the findings presented in Table S2 (See Supplementary Materials).

Based on the regression equation, the optimal conditions for the RSM-optimized BRP extraction process were obtained as A:24.67 (g/mL), B:76.45 °C, C:9.7 min, and D:292 W. The optimal extraction conditions were adjusted according to the actual operation of the extraction process as follows: A:25 mL/g, B:76.5 °C, C:9.7 min, and D:292 W. To verify the accuracy of the predicted results, a validation experiment was conducted. As a result, the actual yield was 21.802 ± 0.682%. In contrast, MUAE has been successfully applied to the extraction of BRP (Table S3, See Supplementary Materials). For example, the extraction temperature was lower and the process was 26.60 times shorter when using MUAE than when using hot water extraction (HWE) for similar BRP yields [7]; moreover, the difference in BRP yields between MUAE and UAE extraction was 15.03-fold, mainly due to the different origins [8].

### 2.3. Polysaccharide Composition

**Figure 3:**
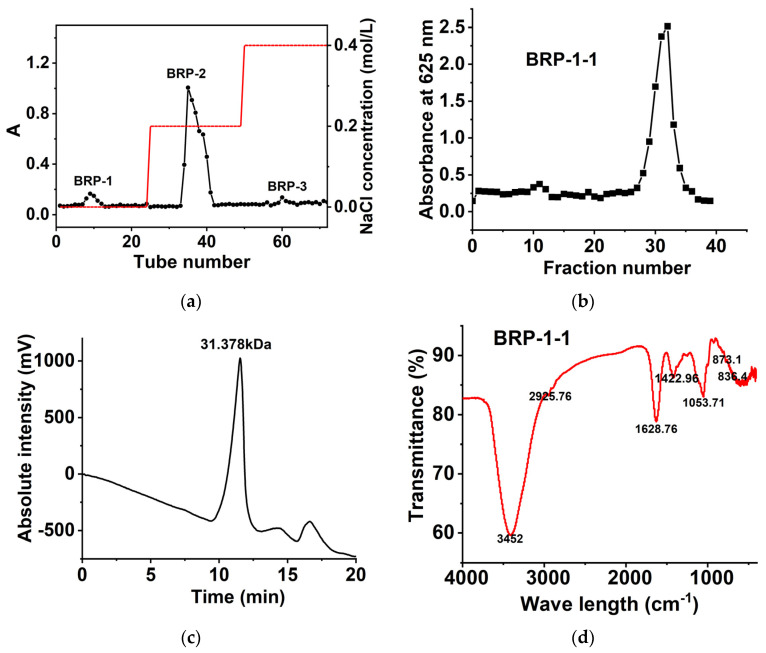
(**a**) Chromatogram of BRP on DEAE-650 column, (**b**) chromatogram of BRP-1 on Sephadex G-100 column; (**c**) the Mw distribution of BRP-1-1; (**d**) the FT-IR spectra of RPP-1-1.

Three polysaccharide fractions, namely, BRP-1 (6.700%), BRP-2 (16.200%), and BRP-3 (2.000%), were isolated from the BRP through the DEAE-650M column (Figure 3a). BRP-1 was purified using Sephadex G-100 to obtain the homogeneous polysaccharide BRP-1-1 (Figure 3b). The total carbohydrate, protein, and uronic acid contents of BRP-1-1 were determined to be 92.346%, 0.670%, and 0.350%, respectively. Furthermore, a single homogeneous peak on the HPLC chromatogram (Figure 3c) was found for the BRP-1-1, and the Mw of BRP-1-1 was calculated to be 31.378 kDa based on the standard curve.

The results of the monosaccharide composition analysis showed that BRP-1-1 consisted of Man and Glu in a molar ratio of 7.62:1. The above results significantly differ from previous reports on BRPs from Urumqi, Xinjiang, China [7], probably because MUAE reduces the BRP molecular weight and thus alters the structures of BRPs.

### 2.4. FT-IR Spectroscopy Analysis

The high absorption peaks of about 3452 and 2925.76 cm^−1^ which can be seen in the FT-IR spectra are caused by the stretching vibrations of -OH and -CH (Figure 3d). The absorption peak generated by water is 1628.76 cm^−1^; the absorption peak generated by the bending vibration of -CH_2_- is 1422.96 cm^−1^. We speculate that the absorption peak of 1053.71 cm^−1^ implies the possible presence of pyranose in the sample [21]. In addition, we found two bands of 836.4 cm^−1^ and 873.1 cm^−1^, which imply that the sugar linkages are α-type and β-type glycosidic bond structures, indicating that BRP-1-1 has a typical polysaccharide absorption peak.

### 2.5. Methylation Analysis

The data related to the total ion rheology of BRP-1-1 are shown in Figure S1 (See Supplementary Materials). The types of the obtained BRP-1-1 are shown in Table 1, including →T-Manp, →2-D-Manp, →2-D-Glup, →4-D-Glup, and →6-D-Glup. This is consistent with the monosaccharide composition analysis result of BRP-1-1.

### 2.6. NMR Analysis

**Figure 4:**
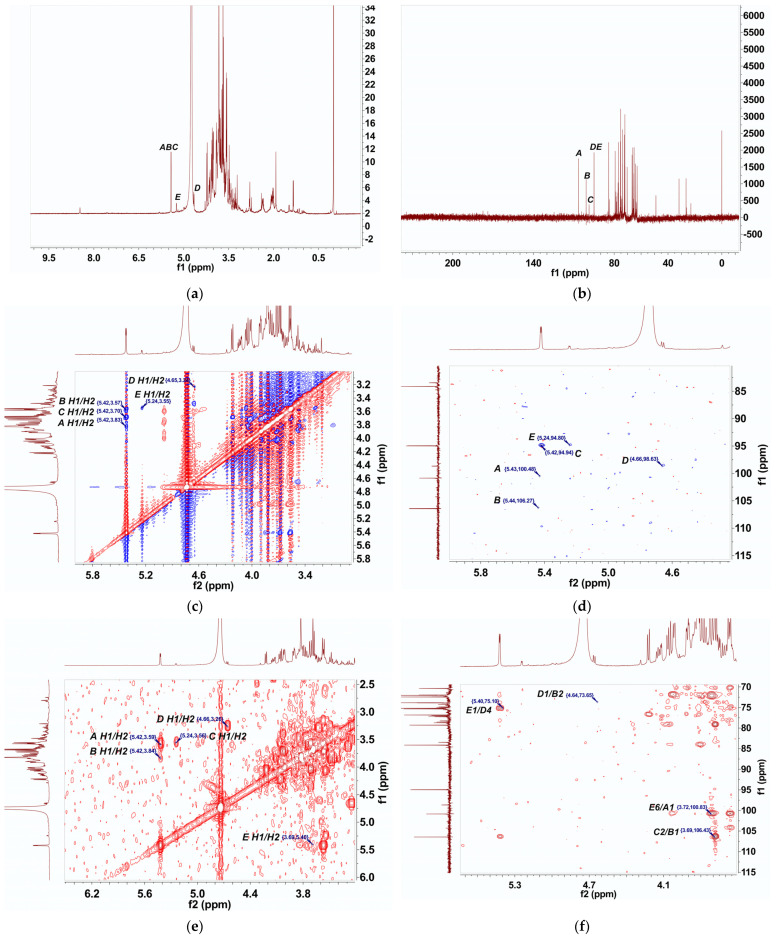

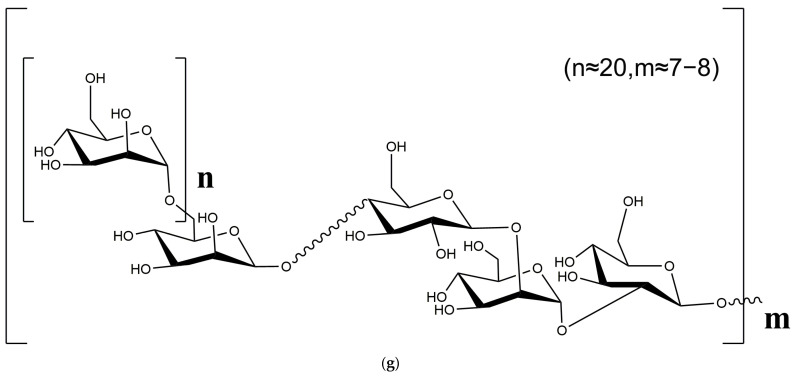
^1^H NMR (**a**), ^13^C NMR (**b**), NOESY (**c**), HSQC (**d**), COSY (**e**), and HMBC spectra of BRP-1-1 (**f**); the predicted structure of BRP-1-1 (**g**).

The NMR spectrum of BRP-1-1 is shown in Figure 4. In the ^1^H NMR spectrum of BRP-1-1 (Figure 4a), three proton signals appear in the heterogeneous proton region. Among them, the signal at 5.24–5.46 ppm was attributed to the α-glycoside conformation; the β-glycoside conformation was characterized with the signal at 4.55–4.68 ppm [22]. Furthermore, in the ^13^C NMR spectrum of BRP-1-1 (Figure 4b), five isomeric carbon signals at 94.93, 94.98, 98.96, 101.98, and 106.86 ppm were labeled as residues A to E, respectively.

We also explored the coupling between proton and carbon signals using HSQC spectroscopy (Figure 4d). By combining methylation analysis, NMR data and the cited literature [23,24], the (H1/C1) cross peaks of residues A to E appeared at (5.43/100.79), (5.43/106.59), (5.42/94.95), (4.66/98.63), and (5.24/94.81), respectively. The above results fully demonstrate that all residues in BRP-1-1 were in α-configuration, except for residue D, which was in β-configuration. This was in strong agreement with the FT-IR results, and the ^1^H and ^13^C chemical shifts are presented in Table S4 (See Supplementary Materials).

The type of substitution of residue D was determined mainly by comparing the chemical shift of the carbon atom in the residue with the corresponding unsubstituted sugar. From the 2D ^1^H-^1^H COSY spectrum (Figure 4e) and the reference data, we could identify other proton signals of 3.24, 3.50, 3.84, 3.47 and 3.71 ppm for residue D (from H2 to H6). The HSQC spectrum (Figure 4d) shows cross peaks for residue D (from H2/C2 to H6/C6) of (3.24/74.88 ppm), (3.50/78.54 ppm), (3.84/74.08 ppm), (3.47/76.83 ppm), and (3.71/62.30 ppm), respectively. It exhibited a chemical shift compared with the C4 signal of residue D, which was not substituted sugar, suggesting that residue D was substituted at C4 [23,24]. Therefore, combining the methylation analysis data and the above NMR results, residue D is considered to be -4-β-D-Glup-(1-. Using a similar approach, we could infer the substitution types of the other residues, obtaining residues A, B, C, and E as T-D-Manp-(1, -2-α-D-Manp-(1-, -2-α-D-Glup-(1-, -6-α-D-Glup-(1-, and -6-α-D-Glup-(1-, respectively. Finally, we combined the complete assignments of the ^1^H and ^13^C chemical shifts of BRP-1-1.

In addition, we needed to assess the HMBC and NOESY spectra of the residues to clarify their sequences [25,26]. In the HMBC and NOESY spectrum (as shown in Figure 4c,f), three cross peaks related to the C1 signal of residue A were observed at (100.86/4.03), (100.86/3.72) and (100.86/3.57), respectively. Among them, 100.86/3.72 ppm indicated the correlation between C1 of residue A and H6 of E. Peaks at 100.86/4.03 and 100.86/3.57 ppm were the C1/H3 and C1/H5 signals of residue A. The cross peaks at 69, 4.66/75.11 and 5.24/74.35 ppm showed that there was a strong correlation between C1 of residue B and H2 of residue C. The H1 of residue D corresponded to the C2 of residue B. The H1 of residue E was also highly correlated with the C4 of residue D. Therefore, based on the above results, the possible predicted structure of BRP-1-1 is [T-α-D-Manp-(1]_n_→6)-α-D-Glup-(1→4-β-D-Glup-(1→2)-α-D-Manp-(1→2)-α-D-Glup-(1→ (*n* ≈ 20, m ≈ 7–8, according to the results of methylation analysis), and the predicted structure of BRP-1 is shown in Figure 4g.

### 2.7. Morphological Properties

**Figure 5:**
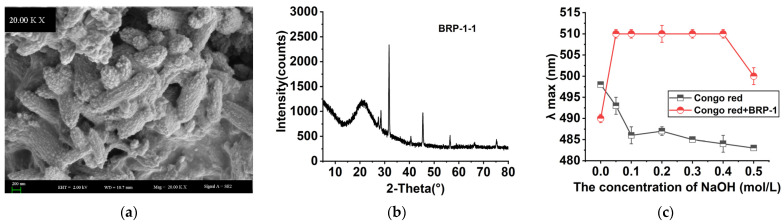
(**a**) SEM of BRP-1-1 observed at 20 KX; (**b**) XRD characterization of BRP-1-1; (**c**) variation curves of λ_max_ of Congo red + BRP-1-1 and Congo red solution.

Purified BRP-1-1 is a highly water-soluble mucopolysaccharide, and its scanning electron micrographs at magnifications of 20 KX are shown in Figure 5a. BRP-1-1 is composed of many pebble-like particles; this is highly different from other kinds of plant polysaccharides in morphological characteristics, which may be due to the different preparation, extraction and purification methods of the product.

### 2.8. XRD Analysis

The XRD pattern of BRP-1-1 is shown in Figure 5b. There is a weak and broad steamed bun peak around 21°, and a sharp and strong diffraction peak near 29°, indicating that there are not only microcrystals in the structure of BRP-1-1, but there is also a polycrystalline system in which crystalline and amorphous coexist [27].

### 2.9. Congo Red Test Analysis

The acid dye Congo red chemically reacts with polysaccharides with a triple helix structure at pH > 7 to produce a Congo red–polysaccharide complex with red-shifted wavelength. When the polysaccharide Congo red complex is in a certain pH range, the complex exhibits a transfer region; the triple helix structure of the polysaccharide is disrupted with increasing pH conditions, which leads to a decrease in the redshift effect of the complex [28].

Figure 5c shows the variation in λ_max_ of the Congo red–polysaccharide complex detected with UV light under the effect of different NaOH concentration gradients. The maximum absorption wavelength of BRP-1-1 for the red shift occurred at a concentration of 0.05 mol/L of NaOH. When the NaOH concentration exceeded 0.4 mol/L, the maximum absorption wavelength of BRP-1-1 decreased, confirming the existence of a triple helix structure of BRP-1-1, which may have anti-cancer and anti-tumor functions to some extent [29,30].

### 2.10. Antioxidant Activity

#### 2.10.1. DPPH Scavenging Rate

**Figure 6:**
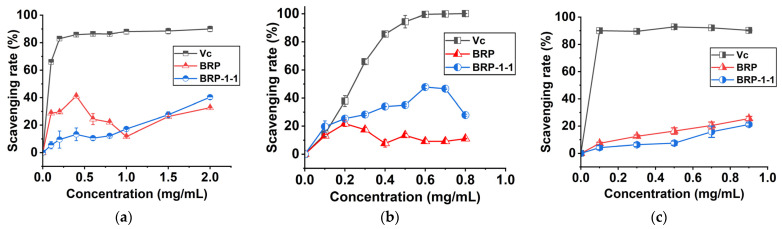
(**a**) DPPH^+^ scavenging rate; (**b**) hydroxyl scavenging rate; (**c**) ABTS^+^ scavenging rate.

In the presence of ethanol, DPPH produces stable nitrogen-containing radicals (purple color in solution) with a characteristic absorption peak at 517 nm [31,32]. Figure 6a shows that the DPPH^+^ clearance activities of BRP, BRP-1-1, and control substances were correlated with concentration, and the scavenging rate varied with different concentrations. The scavenging rate of BRP-1-1 (the IC_50_ value of BRP-1-1 was 2.772 mg/mL), BRP and Vc were 40.313%, 32.697%, and 90.010% at 2.0 mg/mL, respectively. Among them, the DPPH^+^ clearance by BRP-1 was much greater than that reported for BRP-1-1 in HWE (22.370%) [7], which may be because BRP-1-1 possesses a lower molecular weight (1510 kDa) than the latter.

#### 2.10.2. Hydroxyl Scavenging Rate

Hydroxyl radicals are extremely powerful reactive oxygen species that can be produced in the human body, and excess hydroxyl radicals can react with biological macromolecules and harm human health. We found that the hydroxyl scavenging rate of BRP, BRP-1-1 and the reference substances varied at low concentrations, and eventually converged to equilibrium (Figure 6b). In ethanol reaction system, samples with excessive concentrations can induce flocculent precipitation in the system, causing interference [33]. The scavenging rate of BRP-1-1, BRP, and Vc were 47.863%, 9.235%, and 99.590% at 0.6 mg/mL, respectively. Among them, the scavenging rate of ascorbic acid (99.590%) against hydroxyl radicals was only 2.08 times higher than that of BRP-1-1 (47.863%). This finding suggests that BPRs can act as electron or hydrogen donors to scavenge hydroxyl radicals.

#### 2.10.3. ABTS Scavenging Rate

It can be seen that the ABTS scavenging rate of BRP, BRP-1-1, and the control substance (ascorbic acid) was strongly concentration-dependent (Figure 6c). The scavenging rate of BRP-1 (The IC_50_ value of BRP-1-1 was 7.112 mg/mL), BRP and Vc were 25.533%, 21.216%, and 90.244% at 0.9 mg/mL, respectively. This finding suggests that BPRs can act as antioxidants to scavenge ABTS^+^. In conclusion, BRP-1-1 extracted by MUAE with a small molecular weight showed strong antioxidant activity and could be studied in follow-up experiments.

### 2.11. Hypoglycemic Activity

#### 2.11.1. α-Glucosidase Inhibitory Activity

The human intestine contains α-glucosidase and α-amylase, which catalyze the hydrolysis of starch and oligosaccharide (1→4)-glycosidic bonds to produce monosaccharides. Among them, α-glucosidase is secreted by small intestinal epithelial cells, whereas α-amylase is mainly secreted by the salivary glands or pancreas. By inhibiting the activity of these digestive enzymes, the absorption of dietary carbohydrates by the cells of the small intestine can be slowed down, resulting in a lowering of blood glucose. Therefore, the development of inhibitors is considered to be a new strategy for the treatment of type 2 diabetes [34,35].

**Figure 7:**
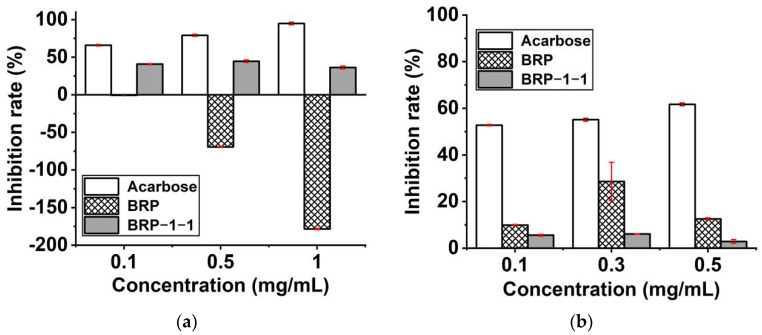
(**a**) α-Glucosidase inhibitory assay; (**b**) α-amylase inhibitory assay.

The inhibitory activity of BRP and BRP-1-1 on α-glucosidase was not concentration-dependent (Figure 7a). At low, medium and high doses, the inhibition of BRP was negative, suggesting that it promotes α-glucosidase to hydrolyze more monosaccharides; at medium doses (0.5 mg/mL), the α-glucosidase inhibition of BRP, BRP-1-1 and acarbose was -69.467%, 44.623%, and 79.100%, respectively. Among them, the considerable variability in the inhibitory activities of BRP and BRP-1-1 may be due to the low molecular weight of BRP-1-1, which has (1→4) and (1→6) glycosidic bonds in its main chain and branched chains, respectively. This is consistent with the results for *Rosa roxburghii Tratt* polysaccharide (RTFP-3); most polysaccharides with hypoglycemic activity have (1→3), (1→4), and (1→6) glycosidic bonds [36]. Furthermore, for the negative values of BRP inhibition, not all plant polysaccharide fractions exhibit hypoglycemic activity [37]; on the other hand, they may be veiled or influenced by polysaccharide components with the same source activity (BRP-1-1). This finding suggests that BRP-1-1 is a potential inhibitor of α-glucosidase.

#### 2.11.2. α-Amylase Inhibitory Activity

The inhibitory activities of BRP and BRP-1-1 on α-amylase were not concentration-dependent (Figure 7b). At low, medium, and high doses, the inhibition rates of both BRP and BRP-1-1 were positive, which indicated that they could both inhibit α-amylase to hydrolyze more starch; at medium doses (0.3 mg/mL), the α-amylase inhibition rates of BRP, BRP-1-1, and acarbose were 28.610%, 6.1%, and 55.120%, respectively. Among them, BRP has higher inhibitory ability than BRP-1-1, which may partly be because BRP has more active components, such as flavonoids, saponins, and alkaloids [38]; it may also be because the glycosidic bond of BRP-1-1 is broken during the reaction. In conclusion, BRP-1-1 exerts a highly hypoglycemic activity.

## 3. Materials and Methods

### 3.1. Materials

*Brassica rapa* L. was planted in Atushi County (39°34′ N, 75°22′ E), Kizilsu Kirghiz Autonomous Prefecture, Xinjiang, China. It was identified by Prof. Shengjun Ma of the Xinjiang Agricultural University. Macroporous resin (AB-8), DEAE-650, Sephadex G-100 and a dialysis bag (molecular weight cut-off: 3.5 kDa) were purchased from Solarbio, China. DPPH and ABTS were purchased from Shanghai Yuanye Biotechnology Co. (Shanghai, China). All reagents were of analytical reagent (AR) grade.

### 3.2. Extraction of Polysaccharides

#### 3.2.1. Extraction Process

The root powder of *B. rapa* L. was refluxed with 95% (*v*/*v*) ethanol 3 times to remove lipids and pigments; 1 g of the powder was taken and added to deionized water with a certain ratio of material. Additionally, polysaccharide extract was obtained after the liquid was extracted in a combined microwave ultrasonic synthesizer/extractor (XH-300A, Beijing Xianghu Technology Development Co., Beijing, China), and suction-filtered before centrifugation. All the extracts were combined and concentrated at 50 °C under reduced pressure with a rotary evaporator. The concentrated solution was precipitated with a 3-fold greater volume of ethanol for 12 h, then centrifuged (8000 rpm, 20 min, 4 °C) and freeze-dried to obtain crude polysaccharide. The crude polysaccharide was dissolved in distilled water and dialyzed for 48 h against deionized water, then treated with macroporous resin (AB-8) to remove colors and proteins, then freeze-dried. The BRP content (%) was determined with the phenol–sulfuric acid technique [22] and calculated as follows:

(2) $$\mathrm{Content}\;\left(\%\right)=\left(\frac{\mathrm{weight of crude BRP}}{\mathrm{weight of B.rapa L root powder}}\right)\times 100\%$$

#### 3.2.2. RSM Experimental Design

Based on the test studies presented in Section 3.2.1, the microwave power was fixed at 440 W, and the effects of ratio of water to material (A: 10–30 mL/g), extraction temperature (B: 70–90 °C) microwave-ultrasound time (C: 3–11 min), and ultrasound power (D: 200–400 W) on the content of BRP were investigated. The experimental optimization design was based on previous studies [16,38]. The complete design comprised 29 experimental points that were executed at random (Table S1, See Supplementary Materials).

### 3.3. Purification of Polysaccharides

After centrifugation, the supernatant of BRP (0.1 g) was sampled on a DEAE-650M cellulose column (4.0 × 20 cm) and gradually eluted with 0, 0.2, and 0.4 M NaCl solution at a flow rate of 2 mL/min. The fraction (18.0 mL) was collected by CombiFlashRF+ (Teledyne Technologies, Inc., Lincoln, NE, USA) and monitored with anthracone–sulfuric acid technique for drawing the elution curve. The fractions were collected according to tube number, then concentrated and lyophilized to obtain polysaccharides. Finally, the BRP-1 (30 mg) was sampled on a Sephadex G-100 column (1.6 × 80 cm) and gradually eluted with distilled water at a flow rate of 0.4 mL/min. The fraction (2.0 mL) was collected according to the conditions mentioned above.

### 3.4. Composition Analysis

The polysaccharide contents, protein contents and uronic acid contents of BRP were measured using the phenol–sulfuric acid technique, bicinchoninic acid (BCA) protein assay, and m-hydroxybiphenyl technique, respectively [39,40,41].

### 3.5. Molecular Weight

The molecular weight (Mw) of BRP was determined based on a previous study [42]. Polysaccharide and standard dextran solutions (1 mg/mL) were filtered through a filter membrane (0.22 μm) and detected using HPLC (Shimadzu Corporation, Kyoto, Japan) apparatus fitted with a column (TSK-GEL-G3000SWXL) and a differential refractive index detector (RID-20A), and then eluted with distilled water at an elution rate of 0.6 mL/min. The standard linear regression equation was used to estimate the Mw of BRP (Mw: 1,5, 25, 50, 100, 250, and 500 kDa).

### 3.6. Monosaccharide Composition

The monosaccharide composition of BRP was also studied based on a previous study [10]. Polysaccharide samples (10 mg) were hydrolyzed with 4 mL trifluoroacetic acid (2M) at 120 °C for 6 h, 3–4 mL of methanol was then added, and rotary evaporation was repeated several times to remove excess TFA. Subsequently, 1 mL chloroform was added to the residue and fully dissolved before filtration (0.22 μm). The pretreated samples were analyzed with gas chromatography–mass spectrometry (7890A-5975C, Agilent Technologies Ltd., Santa Clara, CA, USA).

### 3.7. Infrared Spectral Analysis

The polysaccharide sample was mixed with potassium bromide powder (1:200), crushed and pressed into pellets. FTIR measurements were performed using a Fourier transform infrared spectrophotometer (2600, Shimadzu Corporation, Kyoto, Japan) in the wavenumber range of 4000–400 cm^−1^.

### 3.8. NMR Spectral Analysis

For NMR spectral analysis, 40 mg of BRP-1-1 was dissolved in deuterated water (0.5 mL), and the ^1^H NMR, ^13^C NMR, COSY, HSQC, HMBC, and NOESY spectra were recorded using an AV-400NMR instrument (Bruker Corporation; Billerica, MA, USA).

### 3.9. Methylation Analysis

The methylation analysis of BRP-1-1 was based on a previous study, with slight adjustments [43]. Briefly, the sample was dissolved in 3 mL DMSO overnight. Subsequently, 20 mg of sodium hydroxide and methyl iodide (0.3 mL) were put in the reaction unit, stirred intermittently, and the solution reacted for 3 h in an ice bath and under light. Finally, the reaction was terminated by adding 4 mmol/L Na_2_S_2_O_3_ (1 mL) solution, and chloroform was added to the reaction solution for extraction and drying to obtain methylated polysaccharides. It could then be judged whether the methylation of BRP-1-1 was complete through the disappearance of OH stretching at 3200–3700 cm^−1^ in the FT-IR spectrum. Finally, the resulting product was hydrolyzed by TFA, reduced by NaBD_4_, acetylated with acid anhydride–pyridine, and analyzed for methylation by GC-MS (7890B-7000C, Agilent Technologies Ltd., Santa Clara, CA, USA).

### 3.10. XRD Analysis

The polysaccharide samples were analyzed using an X-ray powder diffractometer (D8, Bruker Corporation; Billerica, MA, USA) at 25 °C. The diffraction angle (2θ) observed at λ = 1.54056A ranged from 5° to 80°, with a rate of 0.1°/s.

### 3.11. Congo Red Test

Equal amounts of 1 mL of Congo red solution (80 μmol/L) and BRP-1-1 solution (1 mg/mL) were mixed. Some NaOH solution and water was added, making the volume constant to 4 mL; the final concentrations of NaOH were 0, 0.05, and 0.1–0.5 mol/L. The solution reacted at 25 °C for 10 min, followed by scanning at 480–520 nm with a UV spectrophotometer (2550, Shimadzu Corporation, Kyoto, Japan).

### 3.12. Scanning Electron Microscopy

The apparent characteristics of BPPS were observed using a SUPRA 55VP scanning electron microscope (SEM). A small amount of sample was fixed uniformly on a thin copper plate, and a layer of gold powder was vaporized on the surface of the sample using an ion sputterer. Subsequently, the polysaccharides were observed under vacuum conditions at 20.0 kV.

### 3.13. Antioxidant Activity Assays In Vitro

#### 3.13.1. DPPH Scavenging Rate

The scavenging rate of DPPH by BRP was based on a previous study [44]. A 0.5 mL mixture of the sample (0.1–2.0 mg/mL) with 1.5 mL of DPPH (1 × 10^−3^ mol/L) was reacted for 30 min at 25 °C under dark condition. The absorbance of the mixture at 517 nm was measured using ascorbic acid (Vc) as a positive control. The percentage clearance (%) was calculated as follows:

(3) $$\mathrm{Scavenging}\;\mathrm{rate}\left(\%\right)=\frac{A_{0}-A_{1}}{A_{0}}\times 100\%$$

where A_0_ and A_1_ are the absorbance of the control group (DPPH + H_2_O) and the test group (DPPH+BRPs/Vc), respectively.

#### 3.13.2. Hydroxyl Scavenging Rate

The scavenging rate of hydroxyl radicals by BRP was obtained based on a previous study [45]. The 0.5 mL sample (0.1–0.8 mg/mL), 0.5 mL FeSO_4_ (9 × 10^−3^ mol/L) solution, and 0.5 mL salicylic acid (9 × 10^−3^ mol/L) solution were mixed thoroughly. Then, 0.5 mL H_2_O_2_ (88 × 10^−3^ mol/L) was added and shaken well before reacting at 37 °C for 30 min. The absorbance of the mixture at 510 nm was measured using Vc as a positive control, and the scavenging rate (%) was calculated as follows:

(4) $$\mathrm{Hydroxyl}\;\mathrm{scavenging}\;\mathrm{rate}=\frac{B_{0}-\left(B_{1}-B_{2}\right)}{B_{0}}\times 100\%$$

where B_0_, B_1_, and B_2_ are the absorbance values of the blank (-OH + H_2_O_2_ + H_2_O), test group (-OH + H_2_O_2_ + BRPs/Vc), and control group (-OH + H_2_O + H_2_O), respectively.

#### 3.13.3. ABTS Scavenging Rate

The scavenging rate of ABTS free radicals of BRP was based on a previous study [46]. For this assay, 1 mL of ABTS (7.2 × 10^−3^ mol/L) solution and potassium persulfate (2.45 × 10^−3^ mol/L) were mixed in equal volumes and reacted at 25 °C for 24 h and under light to produce ABTS free radical (ABTS^+^) solution. Next, ABTS^+^ was diluted with PBS buffer to yield an absorbance of 0.70 ± 0.02 at 734 nm. Then, 1 mL solutions of the samples (0.05–1.0 mg/mL) were mixed well with 9 mL ABTS^+^, and the mixture reacted for 10 min at 25 °C. Finally, Vc was used as a positive control, and the scavenging rate (%) was calculated using Equation (1).

### 3.14. Hypoglycemic Activity Assays In Vitro

To investigate the in vitro hypoglycemic activity of BRPs, their α-glucosidase [16] and α-amylase inhibitory activities [37] were determined by the respective reported methods, with acarbose used as a positive control.

### 3.15. Statistical Analysis

All experiments were carried out three times in parallel, and the data were averaged and statistically analyzed using SPSS (26.0), with a *p*-value < 0.05 indicating statistical significance. RSM analysis was performed here using Design Expert (Version 8.0.6.1). Graphics are drawn using software such as OriginPro 2021b (64-bit) SR2.

## 4. Conclusions

In this study, MUAE was successfully applied for the extraction of BRP. The experimental results showed that using RSM-based optimized MUAE for BRP, with an extraction rate of up to 21.802 ± 0.682%, and isolated and purified a homogeneous polysaccharide BRP-1-1 with an Mw of 31.378 kDa from BRP. BRP-1-1 contains a triple helix structure at the spatial level. It consists of pebble-like particles, which have crystalline and non-crystalline structures; the structure of BRP-1-1 was [T-α-D-Manp-(1]_n_→6)-α-D-Glup-(1→4-β-D-Glup-(1→2)-α-D-Manp-(1→2)-α-D-Glup-(1→. In addition, BRP-1-1 exhibited some resistance to DPPH, and hydroxyl and ABTS radicals exhibited some scavenging activity; it also had a strong inhibitory activity against α-glucosidase. The results suggest that BRP-1-1 extracted from BRP based on MUAE can be used as a natural antioxidant and hypoglycemic for pharmaceutical applications.

## Figures and Tables

**Table 1:** Methylation analysis of BRP-1-1.

Retention Time (min)	Deduced Link-Age	PMAA	Mw	Relative Amount (%)	Major Mass Fragments (*m*/*z*)
8.944	t-Man(p)	1,5-di-O-acetyl-2,3,4,6-tetra-O-methyl mannitol	323	82.611	87,102,118,129,145,162,205,239
13.746	2-Man(p)	1,2,5-tri-O-acetyl-3,4,6-tri-O-methylmannitol	351	3.597	87,102,118,129,162,189,203
24.939	2-Glu(p)	1,2,5-tri-O-acetyl-3,4,6-tri-O-methyl glucitol	351	4.21	84,98,116,145,160,226,267
23.629	6-Glu(p)	1,5,6-tri-O-acetyl-2,3,4-tri-O-methyl glucitol	351	4.869	86,102,115,145,157,187,217
22.29	4-Glu(p)	1,4,5-tri-O-acetyl-2,3,6-tri-O-methyl glucitol	351	4.712	84,98,116,145,160,226,239

## Data Availability

Not applicable.

## Supplementary Materials

The following supporting information can be downloaded at: https://www.mdpi.com/article/10.3390/molecules27093002/s1, Table S1: The BBD design and predicted values for the content of BRP; Table S2: ANOVA for response surface quadratic model; Table S3: Comparison of BRP extraction studies by different methods; Table S4: ^1^H and ^13^C NMR chemical shifts (ppm) for residues of BRP-1-1. Figure S1: Total ion rheology of BRP-1-1.

## Sample Availability

Samples of the compounds are not available from the authors.
