# Supplementary material for: Isolation, Structural Elucidation, Antioxidant and Hypoglycemic Activity of Polysaccharides of Brassica rapa L
Source: Molecules. 2022 May 7;27(9):3002. doi: 10.3390/molecules27093002 (PMC9104227; doi:10.3390/molecules27093002)
Supplement: Supplementary file 1 [file molecules-27-03002-s001.zip › Supplementary Materials.pdf]

Article

Isolation, Structural Elucidation, Antioxidant and Hypoglycemic Activity of Polysaccharides of *Brassica rapa* L.

Wenyang Cao [1], Chenxi Wang [1], Xiayidan Mayhesumu [1], Le Pan [1], Yan Dang [1], Abulimiti Yili [2], Aytursun Abuduwaili [1,*] and Sanawar Mansur [1,*]

[1] College of Chemistry and Chemical Engineering, Xinjiang Agricultural University, Urumqi 830052, China; caoziye88@163.com (W.C.); w18129196969@163.com (C.W.); xiayidanms@sina.com (X.M.); inmail911@sina.com (L.P.); mg20000330@163.com (Y.D.)

[2] Key Laboratory of Plants Resources and Chemistry of Arid Zone, Xinjiang Technical Institute of Physics and Chemistry, Chinese Academy of Sciences, Urumqi 830011, China; abu@ms.xjb.ac.cn

* Correspondence: aytursun11@126.com (A.A.); sanam0405@163.com (S.M.); Tel.: +86-152-7667-9155 (A.A.); +86-139-9921-2592 (S.M.)

Supporting Information

Table S1. The BBD design and predicted values for the content of BRP.

No.	Variables				Yield (%)		
	A: Ratio of water to material (mL/g)	B: Extraction temperature (◦C)	C: Microwave-ultrasound time (min)	D: Ultrasound power (W)	Actual	STDE_V	Predicted
1	20	70	9	300	16.8124	0.0572	16.3700
2	30	70	9	300	17.0450	0.0610	16.2700
3	20	80	9	300	17.5870	0.0458	17.7800
4	30	80	9	300	18.5480	0.0458	18.4100
5	25	75	7	250	17.0603	0.0381	16.8200
6	25	75	11	250	19.3715	0.0153	19.4400
7	25	75	7	350	19.4940	0.5721	18.8400
8	25	75	11	350	18.7920	0.0610	18.4500
9	20	75	9	250	19.0816	0.0763	19.5400
10	30	75	9	250	18.2235	0.0130	17.3200
11	20	75	9	350	16.8850	0.0534	17.5600
12	30	75	9	350	21.0040	0.0458	20.3200
13	25	70	7	300	16.9310	0.0229	16.9400
14	25	80	7	300	16.2136	0.0305	15.7700
15	25	70	11	300	14.8940	0.0839	15.1100
16	25	80	11	300	20.0580	0.0229	19.8200
17	20	75	7	300	18.4370	0.0038	18.2500
18	30	75	7	300	17.9070	0.0610	19.4200
19	20	75	11	300	20.9580	0.0458	20.2600
20	30	75	11	300	18.6240	0.0153	19.6200
21	25	70	9	250	14.8940	0.4500	15.3000
22	25	80	9	250	16.3814	0.0229	16.6000
23	25	70	9	350	14.7500	0.3509	15.3400
24	25	80	9	350	17.1750	0.3966	17.5800
25	25	75	9	300	21.8730	0.0610	21.8700
26	25	75	9	300	21.8730	0.0610	21.8700

27	25	75	9	300	21.8730	0.0610	21.8700
28	25	75	9	300	21.8730	0.0610	21.8700
29	25	75	9	300	21.8730	0.0610	21.8700

Table S2. ANOVA for response surface quadratic model.

Source	Sum of Squares	DF	Mean Square	F -Value	P-value
Model	130.48	14	9.32	15.82	< 0.0001
A- Ratio of water to material	0.21	1	0.21	0.36	0.5593
B- Extraction temperature	9.43	1	9.43	16	0.0013
C- Microwave-ultrasound time	3.69	1	3.69	6.26	0.0253
D- Ultrasound power	0.79	1	0.79	1.35	0.265
AB	0.13	1	0.13	0.23	0.6425
AC	0.81	1	0.81	1.38	0.2596
AD	6.19	1	6.19	10.51	0.0059
BC	8.65	1	8.65	14.68	0.0018
BD	0.22	1	0.22	0.37	0.5512
CD	2.27	1	2.27	3.85	0.0699
A^2	7.77	1	7.77	13.19	0.0027
B^2	82.78	1	82.78	140.48	< 0.0001
C^2	12.55	1	12.55	21.3	0.0004
D^2	28.45	1	28.45	48.29	< 0.0001
Residual	8.25	14	0.59		
Lack of Fit	8.25	10	0.82		
Pure Error	0	4	0		
C.V. %	4.15				
PRESS	47.52				
R^2=0.9405					
R^2_{adj}=0.8811					

Table S3. Comparison of BRP extraction studies by different methods.

Source	Extraction Method	Extraction temperature (◦C)	Microwave-ultrasound time (min)	Ratio of water to material (mL/g)	Ultrasound power (W)	Microwave power(W)	Yield (%)	References
Urumqi, Xinjiang, China	HWE	93.3	258	75	-	-	21.480± 0.410	[1]
Yushu, Qinghai, China	UAE	60.01	55.05	43.05	366.45	-	1.450± 0.110	[2]
Atuh, Xinjiang, China	MUAE	76.5	9.7	25	292	440	21.800± 0.680	This study

Table S4. ^{1}H and ^{13}C NMR chemical shifts (ppm) for residues of BRP-1-1.

Sugar residue		Chemical shift (ppm)					
		H1/C1	H2/C2	H3/C3	H4/C4	H5/C5	H6/C6
A	T-α-Manp	5.42/100.86	3.84/71.91	4.06/76.76	3.72/67.55	3.57/74.30	3.73/63.52
B	1,2-α-Manp	5.42/106.42	3.59/73.88	4.05/74.93	3.72/67.08	3.92/73.78	3.83/61.75
C	1,2-α-Glcp	5.42/94.94	3.69/70.38	3.57/74.30	3.43/72.36	3.84/72.39	3.76/61.8
D	1,4-β-Glcp	4.66/98.63	3.24/74.88	3.50/78.54	3.84/74.08	3.47/76.83	3.68/62.30
E	1,6-α-Glcp	5.24/94.80	3.55/73.83	3.71/75.47	3.42/72.36	3.43/74.15	3.72/72.26

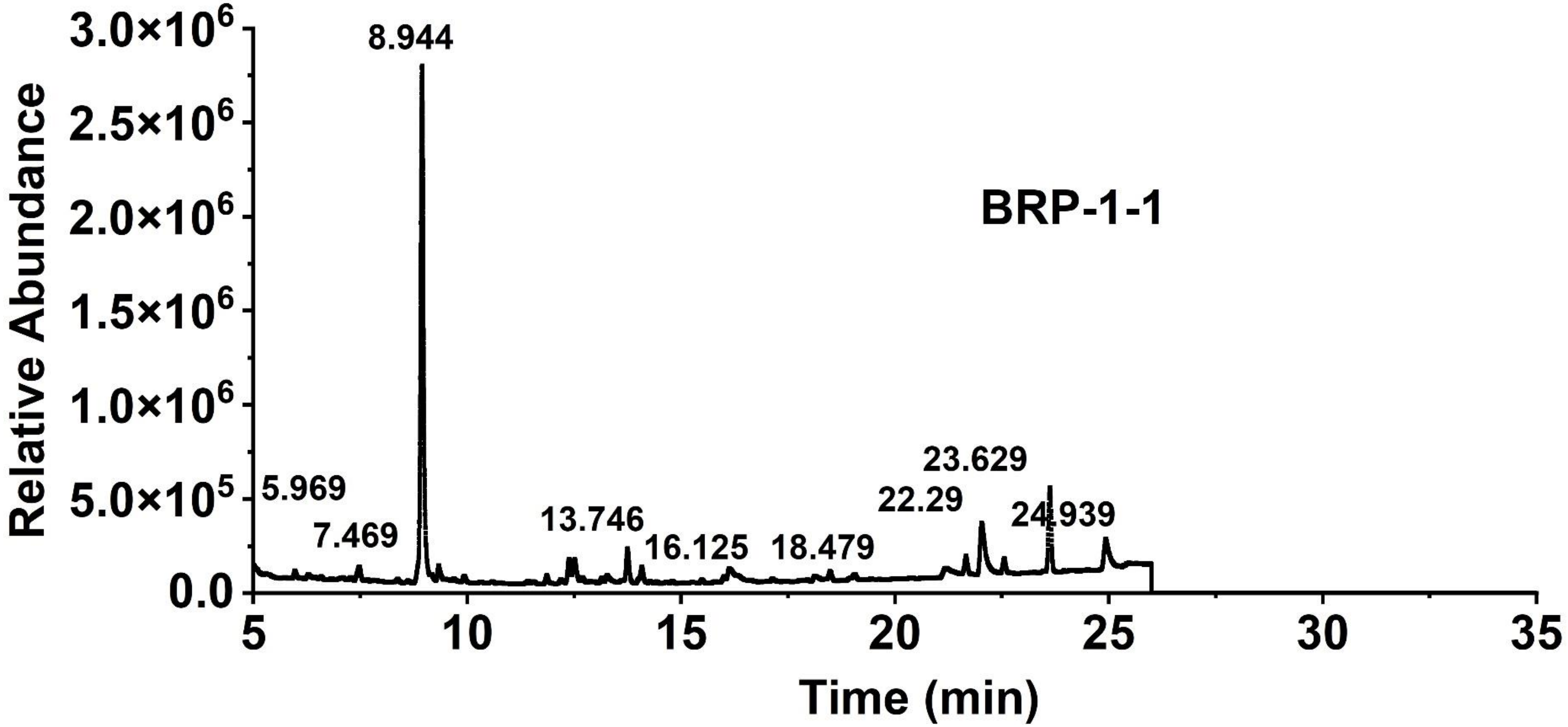

Figure S1. Total ion rheology of BRP-1-1

References

1. Wang, W.; Wang, X.; Ye, H.; Hu, B.; Zhou, L.; Jabbar, S.; Zeng, X.; Shen, W. Optimization of extraction, characterization and antioxidant activity of polysaccharides from *Brassica rapa L. International Journal of Biological Macromolecules* **2016**, *82*, 979-988, doi:https://doi.org/10.1016/j.ijbiomac.2015.10.051.
2. Zhao, W.; Zhang, W.; Liu, L.; Cheng, Y.; Guo, Y.; Yao, W.; Qian, H. Fractionation, characterization and anti-fatigue activity of polysaccharides from *Brassica rapa L. Process Biochemistry* **2021**, *106*, 163-175, doi:https://doi.org/10.1016/j.procbio.2021.04.016.
